# The sustainable professor

**DOI:** 10.7554/eLife.31083

**Published:** 2017-08-17

**Authors:** Elizabeth S Haswell

**Affiliations:** Department of BiologyWashington University in Saint LouisSaint LouisUnited States

**Keywords:** point of view, careers in science, scientific excellence, sustainability, productivity, *A. thaliana*

## Abstract

Responsible agricultural practices provide a useful lens through which to consider the lives and careers of researchers.

Trained and selected for our abilities as researchers, faculty members in the life sciences play an increasing number of roles for which we have little or no training. We are teachers, mentors, managers, writers, editors, peer reviewers, statisticians, fundraisers, accountants, travel agents, recruiters, conference organizers, small business owners, science communicators, graphic designers, web designers, ethics compliance monitors, project managers, data storage and sharing experts, political activists, science advocates, public speakers, science outreach specialists, public relations gurus, mental health monitors, mediators, cheerleaders, life coaches, career counselors, therapists, immigration consultants, role models, and social directors… just to mention a few. For the most part, we are eager to work in these different capacities, and many of them are intrinsic to successful research careers. I can say from personal experience that stretching one’s skill set can be enormously invigorating. However, as we take on new duties without removing old ones, our time and attention – which are not infinite – are being split into smaller and smaller pieces.

Our working hours have expanded as well. The omnipresence of email, team communication apps and messaging systems blur the line between work and home life to the point where borders no longer exist. This allows our work to demand as much of our time as we are willing to give – and the pervading scientific culture, at least in the US, dictates that we give essentially *all* of our time. Proofs with a 24-hour window to reply, requests to edit or review manuscripts, messages about department business or committee work all arrive – not just into our inbox but into our consciousness – at any hour of the day, any day of the week. The struggle to completely leave our jobs for a weekend, never mind a week of vacation, is real. And this is a pity, because arguably the most valuable resources we have as life scientists are our time and our intellect, and they are intimately connected. The sharpest mind in the world still requires time dedicated to careful thinking, reading, planning, and allowing ideas to percolate.

I believe that increases in the number of roles that faculty members play and in the time we spend on our work are endangering our ability to produce new ideas and effectively mentor new scientists. Furthermore, our attitudes and the examples we set will select for the next generation of academic researchers. If we insist not only that this is all doable, but that it all *must* be done, we run the risk of losing great minds and great personalities from our profession. If we genuinely want to encourage diversity in our ranks, we need to figure out how to allow for variations in inherent skills, passions, priorities and energies. Below, I argue that solutions to these problems may be found by considering three concepts from sustainable agriculture.

## Inspiration from the sustainable agriculture movement

Sustainable agriculture was conceived as a necessary response to an industrial farming system that expects continuous growth, values current productivity at the expense of future output, and seems based on the idea that natural systems are to be overcome rather than honored (Stauber, 1995). These industrial agricultural practices often deplete topsoil, pollute water, and affect the well-being of farmworkers (DeLonge et al., 2016). In contrast, the goal of sustainable agriculture is to ensure that we meet the needs of the current world population in a way that takes into account the needs of future generations. It employs agricultural practices that promote the livelihood of farmworkers and protect nutritious topsoil and clean water sources in order to ensure the future productivity of the farm and its surrounding ecosystem (Feenstra et al., 2017; Tilman et al., 2002).

Like the topsoil on a farm, a scientist’s ability to produce thoughtful and creative teaching, research and service must be protected in order to ensure long-term productivity and to prevent damage to the ecosystem. Too often we sustain constant productivity with the inorganic fertilizers of coffee and wine, and generate toxic run-off like negativity, competition and self-doubt. (I leave for a different essay the topic of pathogenic publishing practices). Below I list a few ways we may stop “depleting the topsoil,” but hope that this is just the beginning of a longer conversation about creating truly sustainable academic lives, institutions and research communities.

Too often we sustain constant productivity with the inorganic fertilizers of coffee and wine

### Biodiversity

One approach to sustainable agriculture is the planting of diverse, naturally evolved and high-yielding species. Scientists also want to be part of a diverse community that taps into people’s natural skills to produce the innovations and insights that will meet the challenges of the future. Yet, we continue to propagate a kind of faculty monoculture where everyone must excel at every aspect of the job. Perhaps we should stop expanding our job description – and in fact should make an effort to contract it. I propose that we can meet the needs of our many stakeholders (students, the public, funding agencies, institutes and so on) as a group, rather than as individuals. For example, a department made up of professors each allowed to focus on teaching, research or service according to their own choice might be more effective than one where all of these activities must be bundled into each individual. By allowing people to pursue their passions – and, importantly, by encouraging everyone to value different kinds of departmental contributions – we could assemble a more diverse and more innovative faculty that serves everyone better and more efficiently.

We can also encourage universities to acknowledge and address the sheer volume of tasks assigned to life sciences faculty. Many departments (but definitely not all!) provide support or take over some roles, including science communication, travel administration and ethics training. Of course, this requires access to a budget and additional administrative personnel, but support for this kind of “shadow work” (Lambert, 2011) might improve efficiency, productivity and faculty happiness so much that it would end up paying for itself.

Funding agencies could help as well. For example, while it is obvious that the work we do should be communicated to the public, it is not obvious that university faculty members are the best ones to do it. What if funders such as the National Science Foundation took the money assigned to “broader impacts” activities in individual grants, and spent it on programs run and executed by staff directly trained in public outreach and education? Other mandates like providing for data storage, mentorship plans and ethics training are indisputably important but their implementation takes time and attention, especially if they are to be accomplished well. Funding agencies could use a portion of their budget to provide services, support or assistance whenever new responsibilities are added to the role of Principal Investigator.

### Crop rotation

A second well-known approach to sustainable agriculture is multi-crop rotation, where soil depletion is avoided by cycling between crops that have different nutrient demands. Similarly, cycling between work and rest is critical for intellectual nourishment and long-term productivity (Jabr, 2013). We are all familiar with the advice to make time for friends, family and hobbies, but it can be very hard to put into practice. It is enormously tempting to immediately start a new task as soon as one is finished, or to check email on vacation. Perhaps we would do better to accept the natural ebb and flow of a project, paper or class. I do not expect that faculty will immediately stop working outside the 9–5 Monday–Friday working week upon reading this essay – but at a minimum we can avoid propagating these expectations to our trainees by not asking them to participate in email or other exchanges during the weekend or late at night. (I need to take my own advice here, and have started using a small application add-on (https://smallcubed.com/mao/) that makes it possible to compose emails at any time, but delay sending them until work hours). Even small steps like these count as we work towards a climate that celebrates rest and recovery as much as hard work and productivity.

To create a sustainable work life, we need to both know our limits and respect them

### Small-scale farming

A third approach to sustainable agriculture is to embrace and protect smaller farms, where more attention can be paid to protecting the ecosystem while still operating with the same efficiency as an industrial-sized farm. Correspondingly, we may wish to scale down our endeavors in order to lead sustainable faculty lives. Saying “no” to a new invitation or idea is easier when it is something that distracts us from our main goals, whatever they may be. However, when it comes to things we love and consider part of our personal mission – say, teaching students, funding new projects, or talking about our group’s research – we tend to assume more is always better. Unfortunately, this is not always the best approach to a consistently productive, creative and fruitful career. To create a sustainable work life, we need to both know our limits and respect them. A wise approach may be to set time aside at the beginning of a semester or year to draw the boundaries that will protect our most important personal and scientific missions. For example, one might limit travel, the number of summer trainees, or new research projects started.

## Summary

Three concepts associated with sustainable agriculture can be applied to the life of biology faculty members, providing yet another illustration of the ways in which the field of plant biology can teach us about ourselves. By moving beyond expectations that each of us needs to be everything to everyone, by acknowledging the important role played by rest in promoting innovation, and by confronting our natural tendency to assume that all growth is good, we may become better stewards of our lives and spirits, deliberately maintaining them for future productivity.
